# From Silent to Severe: Gastric Perforation Causing Spontaneous Hydropneumothorax Secondary to a Large Hiatal Hernia

**DOI:** 10.7759/cureus.61518

**Published:** 2024-06-02

**Authors:** Sai Rakshith Gaddameedi, Jayasree Ravilla, Anoohya Vangala, Malay Rathod, Ojas Chinchwadkar, Montaser Alrjoob, Vandana Bandari, Doantrang Du

**Affiliations:** 1 Internal Medicine, Rutgers Health/Monmouth Medical Center, Long Branch, USA; 2 Internal Medicine, Rutgers Robert Wood Johnson Medical School, New Brunswick, USA; 3 Internal Medicine, Bayhealth Medical Center, Dover, USA

**Keywords:** paraesophageal hiatal hernia, hiatal hernia, hydropneumothorax, pleural effusion, gastric perforation

## Abstract

Hiatal hernias, characterized by the protrusion of internal organs through the diaphragmatic hiatus, are commonly seen in the elderly age group. While surgical management remains debatable for asymptomatic cases, emergent complications necessitate prompt intervention. Here, we present a case of a 69-year-old female with a history of diaphragmatic hernia, who developed acute hypoxic respiratory failure secondary to acute pleural effusion caused by paraesophageal hernia rupture. Despite initial inconclusive imaging, a CT scan revealed the severity, prompting emergent management. The patient underwent esophageal stent placement, video-assisted thoracoscopic surgery-assisted total lung decortication, and three chest tubes placement, followed by antimicrobial therapy. Favorable outcomes were achieved with multidisciplinary intervention, highlighting the importance of timely recognition and comprehensive diagnostic approaches. This case underscores the potential severity of hiatal hernias, particularly paraesophageal types, necessitating vigilance among clinicians for timely intervention. It also emphasizes the effectiveness of combined surgical and medical multidisciplinary approaches in such emergent situations for optimal patient outcomes.

## Introduction

A hiatal hernia occurs when internal organs, such as the stomach, protrude through the hiatus of the diaphragm. It can lead to laxity of the lower esophageal sphincter and reflux of gastric contents into the esophagus, causing gastroesophageal reflux disease (GERD). The prevalence of hiatal hernias in the United States is 10-20%, and between 55% and 60% of individuals over the age of 55 years are afflicted [1]. Additionally, women tend to be affected more frequently than men. The likelihood of acquiring a hiatal hernia can be raised by variables like heredity, smoking, weight, and pregnancy. Hiatal hernias can also form due to persistent intra-abdominal pressure, which can occur from heavy lifting or straining during bowel motions [2]. There is ongoing debate on the indications of surgical management of asymptomatic or minimally symptomatic paraesophageal hernias. In recent decades, medical management of paraesophageal hernias has improved, and the risks of emergent procedures have declined, leading many clinicians to support a conservative approach to management [3]. Occurrences of rare complications like incarceration and perforation require emergent surgery [4]. Here, we present a case where the rupture of a paraesophageal hernia led to acute pleural effusion, shortness of breath, and rapid clinical deterioration.

## Case presentation

**Figure 1:**
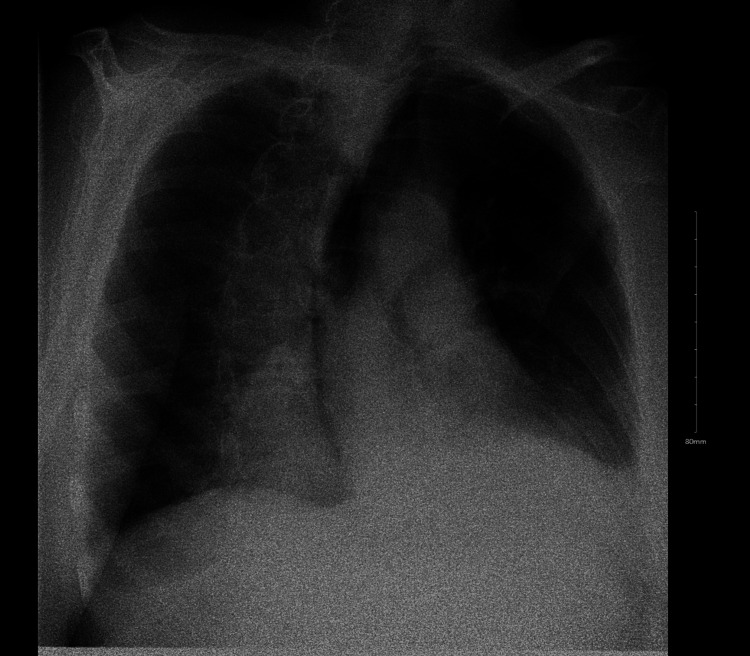
Fluoroscopic upper gastrointestinal series with single contrast showing clear lung fields.

A 69-year-old female with a history of hypertension, anxiety, and diaphragmatic hernia status post laparoscopic Nissen fundoplication four years ago presented to the emergency department with complaints of nausea, vomiting, and epigastric pain for four days. She was in her usual state of health until she started having progressively increasing nausea, followed by nonbilious and non-bloody vomiting, associated with dull epigastric pain and constipation. The patient had been having these symptoms on and off over the past two months, but her symptoms had worsened over the past four days to the extent that she could not tolerate oral intake. Physical examination revealed no abdominal guarding, rigidity, or distension. Her blood work revealed an unremarkable complete blood picture, comprehensive metabolic panel, troponin, and lactic acid. An abdominal ultrasound showed no abnormal pathology. Computed tomography (CT) of the abdomen and pelvis without contrast was done outpatient three days ago to evaluate her abdominal pain, showing a large type 3 paraesophageal hernia with an under-distended distal stomach without volvulus. The patient was admitted for further workup. A fluoroscopic upper gastrointestinal series with a single contrast (Figure 1) was done to evaluate the tightness of the wrap placed four years ago, and no evidence of obstruction or extraluminal contrast leakage was found.

**Figure 2:**
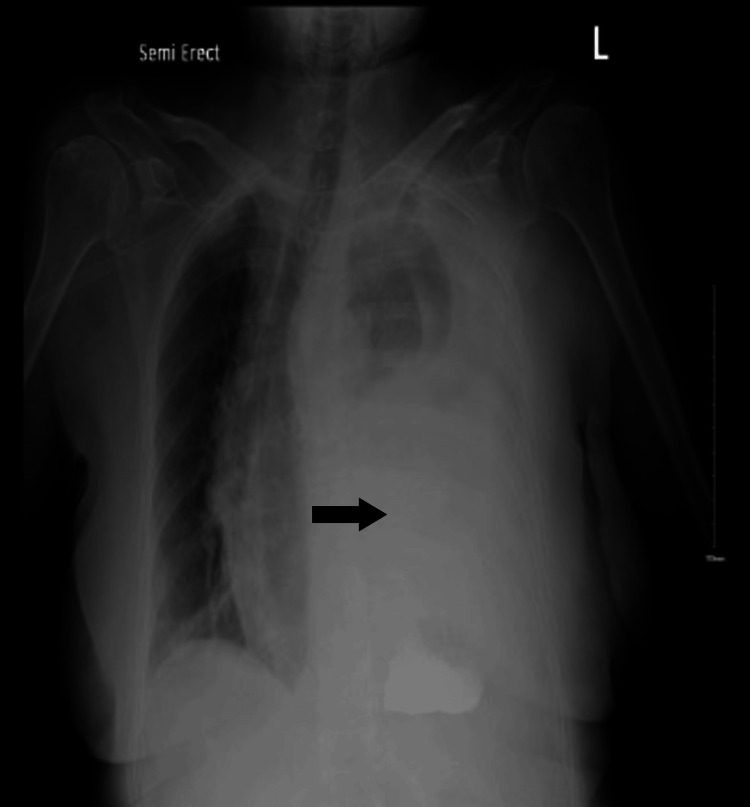
Chest X-ray revealing large left-sided pleural effusion.

**Figure 3:**
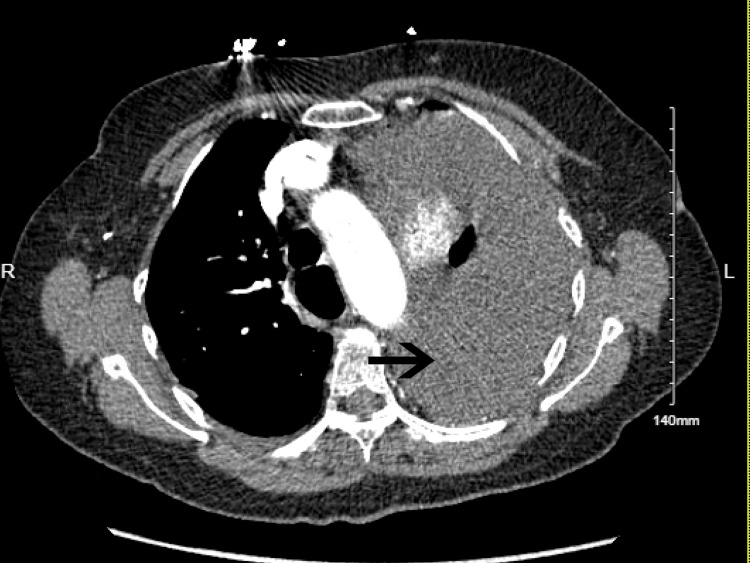
CT angiography of the revealing large left-sided hydropneumothorax.

The following day, the patient started having severe back pain and became hypoxic, requiring a non-rebreather mask with absent breath sounds on the left hemithorax. Chest X-ray (Figure 2) showed left-sided pleural effusion with tracheal deviation to the right side. Given the suspicion of gastric/hiatal hernia perforation causing acute pleural effusion, the patient was transferred to the intensive care unit. An emergent chest tube was placed at the bedside after the CT chest (Figure 3) revealed a large left-sided hydropneumothorax.

**Figure 4:**
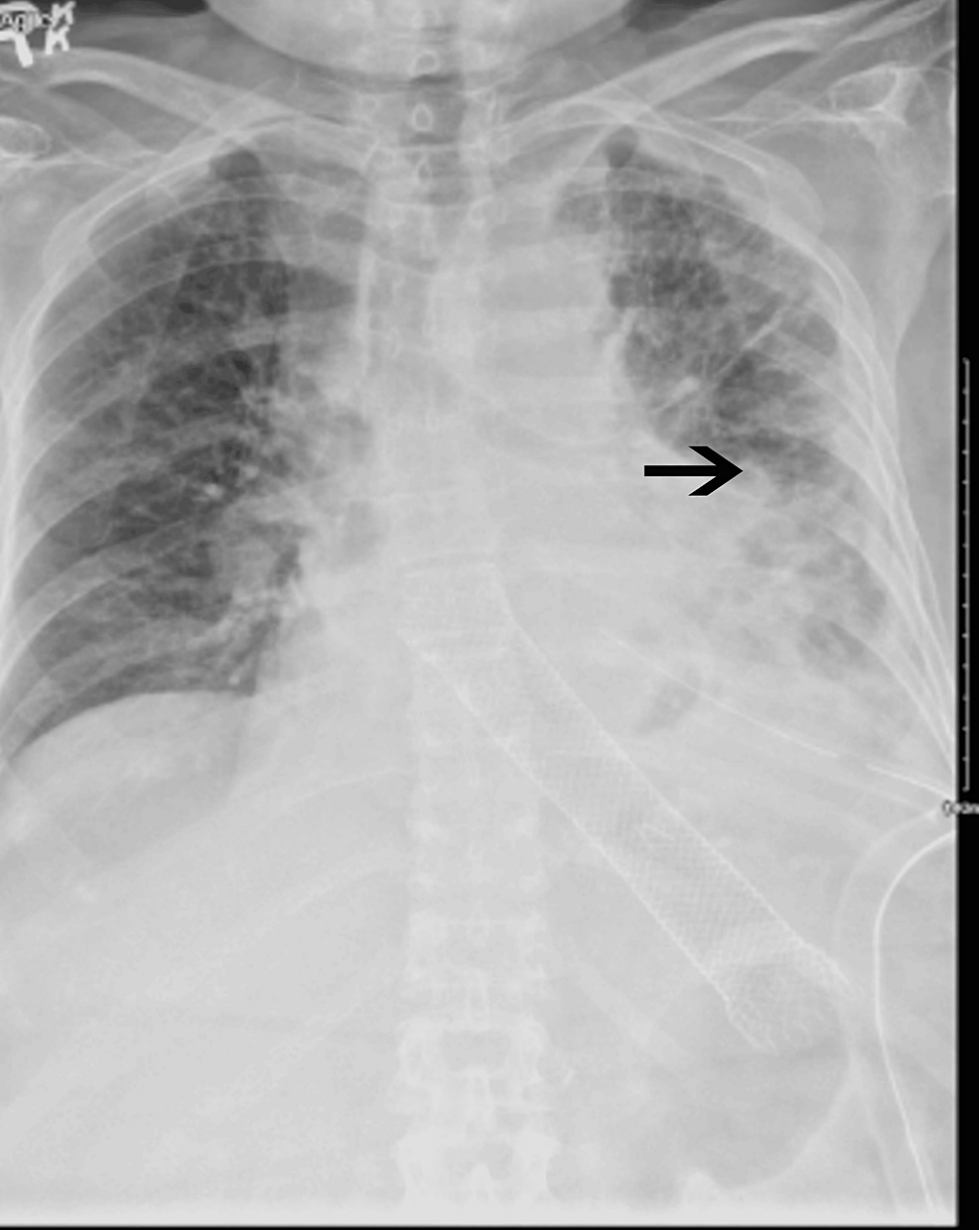
Repeat chest X-ray post surgery showing improvement in left-sided pleural effusion.

**Figure 5:**
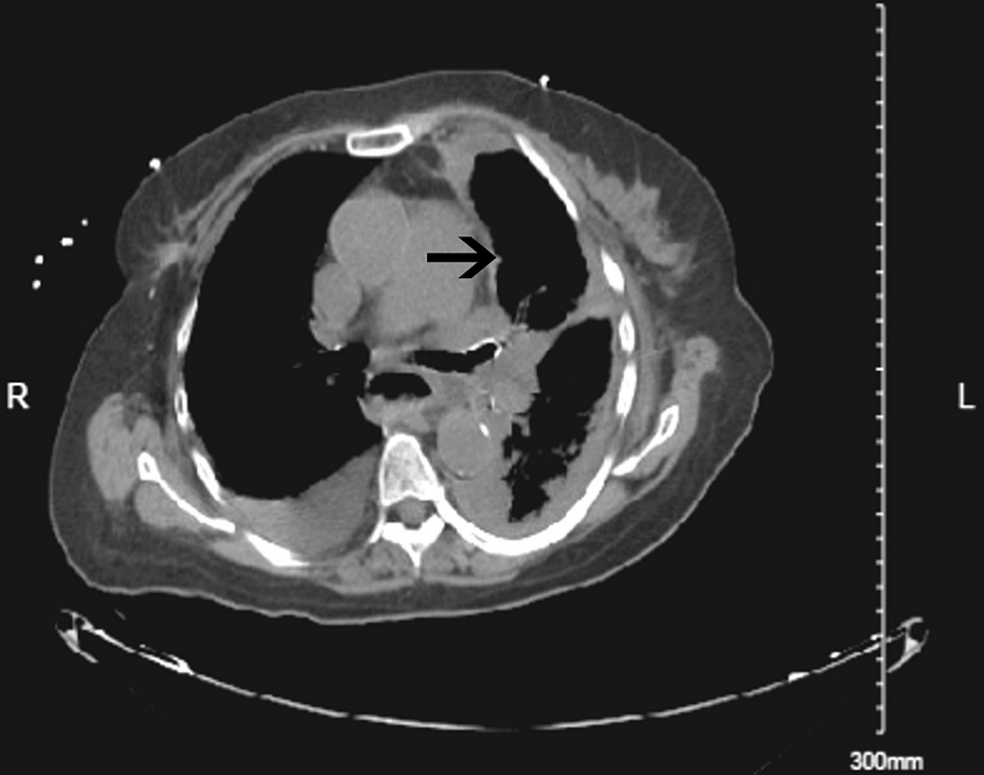
Repeat CT of the chest post surgery showing improvement in left-sided pleural effusion.

The chest tube drained 1.7 liters of blood-tinged fluid, which was sent for fluid analysis and amylase level. The patient was taken for emergent esophagogastroduodenoscopy (EGD) to evaluate for possible gastric rupture. The patient was intubated before the procedure. Endoscopy showed incarcerated herniation of the posterior gastric fundus into the left chest with obstruction, gangrene, and left hydropneumothorax. A perforation was noted in the area of the esophagus opening into the portion of the stomach, which herniated into the chest. An esophageal stent was placed to cover this area. A total lung decortication was performed with the placement of three chest tubes and a J tube. After that, a left paraumbilical incision was made, and gastric resection along with the freeing up of the hiatal hernia was performed using a laparoscopic approach. The patient was then transferred back to the ICU on mechanical ventilation and vasopressor support. Pleural fluid analysis showed an elevated amylase of 8601 U/L, and cytology showed candida species with marked acute inflammation. Her left lung cultures from the operating room also had heavy growth of candida albicans along with a few gram-positive bacilli. Hence, the patient was started on antimicrobial coverage with intravenous fluconazole and piperacillin-tazobactam. The patient was gradually weaned off the ventilator and vasopressors. She subsequently got extubated three days after the procedure and started tolerating oral intake. Eventually in the next two weeks, all the chest tubes were removed. The biopsy report from EGD showed gastric necrosis with perforation, and the organisms were consistent with candida. The patient completed a 14-day course of antifungals and antibiotics. A repeat chest X-ray (Figure 4), CT of the chest (Figure 5), and esophagogram were performed before discharge to home, showing significant improvement.

## Discussion

Hiatal hernias are classified into four types. In type I, known as sliding type hernias, the gastroesophageal junction (GEJ) is displaced upward toward the hiatus, accounting for approximately 95% of cases [5]. Type II, or paraesophageal hiatal hernias, occurs when a portion of the stomach moves alongside the esophagus into the mediastinum. Type III involves a sliding hernia and paraesophageal hernia, where both the stomach and the GEJ protrude into the mediastinum. Type IV is characterized by the herniation of the spleen, colon, or small intestine into the chest cavity and the stomach [5]. Patients with hiatal hernias commonly experience respiratory complications due to chronic GERD, ranging from temporary dyspnea to life-threatening bronchoconstriction and acute respiratory failure. Aspiration of gastric and esophageal contents can result in bronchial irritation and aspiration pneumonia, presenting with imaging findings like bronchiolitis and lung opacities [1]. Diminished oxygen saturation in patients with hiatal hernias suggests pulmonary manifestations of extensive GERD, especially in type IV hernias [6]. Direct compression of mediastinal structures by large hiatal hernias may contribute to cardiopulmonary complications [7]. Large paraesophageal hernias can lead to gastric volvulus, which may cause dysphagia, postprandial pain, ischemia, and, in rare instances, strangulation. Gastric ulceration, gastritis, or erosions within the incarcerated hernia pouch can rarely cause bleeding [8]. In our case, the patient with a history of paraesophageal hernia underwent Nissan's fundoplication for correction. While effective, revisions may be necessary in some cases. Our patient developed symptomatic incarcerated, gangrenous type III paraesophageal hernia, which was managed with esophageal stent placement. Studies reveal incidentally found asymptomatic diaphragmatic/hiatal hernias, initially without signs of obstruction but later presenting with blood extravasation from stasis due to strangulation, affecting up to 0.17% of the adult population [9].

Epigastric pain poses diagnostic challenges with diverse cardiothoracic and intra-abdominal possibilities, seldom including a perforated hiatus hernia in differentials [10]. Comprehensive blood tests, cardiac enzymes, amylase, blood gases with lactate, and ECG aid in ruling out alternative diagnoses [11]. It can be challenging to diagnose a late-presenting diaphragmatic hernia. Auscultation of the chest reveals audible bowel sounds, which may indicate dislocated intestinal loops. While a chest X-ray may offer initial insights, our case presented uncertainties, lacking pneumoperitoneum or visible hernia, but it revealed a large left-sided pleural effusion [12]. A definitive diagnosis of hiatal hernia rupture in our case was established through CT findings, emphasizing its crucial role when the clinical urgency is recognized. Still, uncertainty persists regarding diagnosis and subsequent management. Timely detection using contemporary imaging tools is essential. In our case, hemothorax was due to the perforation of a gangrenous hiatal hernia segment. The patient was promptly transferred to the medical intensive care unit, where the emergency was managed initially by placing a bedside chest tube and later with esophageal stent placement and video-assisted thoracoscopic surgery (VATS)-assisted total lung decortication. This intervention resulted in a notable decrease in pleural effusion, as confirmed by a post-intervention chest X-ray. Paraesophageal hiatal hernias often recur at a high frequency. Despite the possibility of such complications, laparoscopic repair is still considered the preferred treatment approach and is typically well-tolerated [13]. This case shows the importance of a multidisciplinary approach by cardiothoracic surgeons, gastroenterologists, infectious disease physicians, and intensivists in handling such situations. Also, it highlights a serious problem with a sliding hiatus hernia, emphasizing the importance for both surgeons and medical professionals to remain vigilant and contemplate this diagnosis when patients show symptoms like chest pain, stomach pain, vomiting, and difficulty breathing.

## Conclusions

This case underscores the potential severity of hiatal hernias, particularly paraesophageal types, which can lead to catastrophic complications such as rupture and subsequent acute pleural effusion. Despite the prevalence of hiatal hernias, diagnosis can be challenging, especially when symptoms mimic other cardiothoracic and intra-abdominal conditions. Utilizing comprehensive diagnostic approaches, including advanced imaging modalities like CT, is crucial for timely recognition and intervention.

Additionally, prompt management, involving a multidisciplinary team, is imperative in such emergent scenarios. Our patient's case illustrates the successful application of a combined surgical and medical approach, including esophageal stent placement, VATS-assisted total lung decortication, and antimicrobial therapy, resulting in favorable outcomes and eventual recovery.
